# A Collection of Components to Design Clinical Dashboards Incorporating Patient-Reported Outcome Measures: Qualitative Study

**DOI:** 10.2196/55267

**Published:** 2024-10-02

**Authors:** Anja Yvonne Bischof, David Kuklinski, Irene Salvi, Carla Walker, Justus Vogel, Alexander Geissler

**Affiliations:** 1 Chair of Health Economics, Policy and Management School of Medicine University of St. Gallen St. Gallen Switzerland

**Keywords:** clinical dashboards, design components, patient-reported outcome measures (PROMs), patient-reported outcomes (PROs)

## Abstract

**Background:**

A clinical dashboard is a data-driven clinical decision support tool visualizing multiple key performance indicators in a single report while minimizing time and effort for data gathering. Studies have shown that including patient-reported outcome measures (PROMs) in clinical dashboards supports the clinician’s understanding of how treatments impact patients’ health status, helps identify changes in health-related quality of life at an early stage, and strengthens patient-physician communication.

**Objective:**

This study aims to determine design components for clinical dashboards incorporating PROMs to inform software producers and users (ie, physicians).

**Methods:**

We conducted interviews with software producers and users to test preselected design components. Furthermore, the interviews allowed us to derive additional components that are not outlined in existing literature. Finally, we used inductive and deductive coding to derive a guide on which design components need to be considered when building a clinical dashboard incorporating PROMs.

**Results:**

A total of 25 design components were identified, of which 16 were already surfaced during the literature search. Furthermore, 9 additional components were derived inductively during our interviews. The design components are clustered in a generic dashboard, PROM-related, adjacent information, and requirements for adoption components. Both software producers and users agreed on the primary purpose of a clinical dashboard incorporating PROMs to enhance patient communication in outpatient settings. Dashboard benefits include enhanced data visualization and improved workflow efficiency, while interoperability and data collection were named as adoption challenges. Consistency in dashboard design components is preferred across different episodes of care, with adaptations only for disease-specific PROMs.

**Conclusions:**

Clinical dashboards have the potential to facilitate informed treatment decisions if certain design components are followed. This study establishes a comprehensive framework of design components to guide the development of effective clinical dashboards incorporating PROMs in health care practice.

## Introduction

Technological advances have created a multitude of innovative prevention and treatment options in medicine. Despite providing benefits to patients, this evolution created settings of increasingly complex medical decision-making [1] and an increased distancing of physicians from their patients [2]. The use of clinical dashboards can support physicians in dealing with various relevant sources of information, such as clinical parameters or patient-reported outcomes (PROs). A clinical dashboard can serve as a data-driven clinical decision support tool if capable of querying multiple data sources and visually representing key performance indicators in a single frame. The added value of a dashboard comes from its ability to provide a concise overview of key information along the treatment process [3]. Theoretically, visualizing data on clinical dashboards should decrease time spent on data gathering, cognitive overload, and time to task completion and should improve situation awareness and compliance with evidence-based safety guidelines [4]. However, the frustration of physicians increases as systems are not running smoothly and sometimes even hinder physicians in their daily work [5].

Incorporating the patient perspective into clinical dashboards by patient-reported outcome measures (PROMs) offers considerable potential to enhance clinical outcomes and patient’s health-related quality of life (HRQoL) [6-9]. Furthermore, it can result in a more patient-centered treatment approach and improved care processes [10,11]. PROMs assess, for instance, patients’ HRQoL, functional impairments, and disabilities. In addition, PROMs can report the nature and severity of symptoms in a standardized way [11,12], independent of physicians’ or caregivers’ interpretation [13,14]. The use of PROMs in clinical dashboards has, thus, been shown to support physicians’ understanding of how treatment impacts symptom scores [11] and to help identify HRQoL deterioration at an early stage [12].

Research on the development of dashboards, including PROMs, has been conducted for outpatient treatment of prostate cancer, overactive bladder, multiple sclerosis, and various surgical procedures [12,15-18]. Furthermore, most of the existing literature has focused on disease-specific PROMs [9,11,16,19,20]. Accordingly, the potential of incorporating a generic PROM, such as the EQ-5D, into a dashboard has not been investigated yet. Incorporating a generic PROM enables comparability across diseases and an overall assessment of the patient’s HRQoL. However, research shows that physicians will only use clinical dashboards if they perceive an additional value [11].

Current literature focuses considerably more often on dashboards for chronic diseases [11,12,16,17,20-22] compared with dashboards on 1-time acute care interventions [18,23]. Furthermore, the literature reveals the relevance of complementary data as important design components of clinical dashboards incorporating PROMs. Components such as patient information (such as demographic information or most recent health updates) [20,24,25] or clinical data (such as lab results and medication data) [20,24,26,27] are highlighted. Furthermore, the inclusion of additional design components such as past assessment scores (ie, the visualization of PRO scores over time) [9,11,27,28], peer-group comparisons (ie, analyzing a patient’s PRO scores with a peer group) [17,27,29], PRO-related and overall health-related goals (ie, goals concerning PROs or overall health developed during patient-physician interaction) [16,27], alerts (ie, in case of critical values) [9,12], free-write in features (ie, additional space for physician’s notetaking) [9,28], and dashboard customizability (ie, degree of how far individual wishes of users may be respected) [16,17,24] are promoted (for more information, please refer to Multimedia Appendix 1).

However, due to the literature’s inconsistency on design components for clinical dashboards, we aim to develop a guide on which design components to consider when building clinical dashboards incorporating PROMs. Therefore, we investigate the following 2 research questions: (1) What are the design components of clinical dashboards incorporating generic and disease-specific PROMs? (2) Do the design components for clinical dashboards incorporating PROMs differ for acute conditions with a 1-time intervention versus chronic conditions?

## Methods

### Overview

We conducted interviews with software producers and users, that is, physicians, to identify design components for user-friendly clinical dashboards to enhance the quality of care [18]. By interviewing 2 stakeholder groups, we collected insights on 2 key perspectives (software producers vs users).

The recruitment of the interviewees followed a consecutive process: first, we recruited in our own network, and then, through a snowballing technique, we were able to contact software producers and users outside of our network. The conduction of interviews ended once thematic saturation was reached, that is, when no additional information was gained from further interviews [30]. The interviews were semistructured, and findings from a previous literature search (see Multimedia Appendix 1) guided the development of the interview questionnaire. Initially, AYB developed the interview guide, while IS, DK, and AG collaborated to refine it. To finalize the questionnaire, a pilot interview was conducted with a key opinion leader in this subject area. After the first 5 interviews, we iteratively tailored the interview structure to align with the evolving interview dynamics, facilitating a focused exploration of our research priorities. This adaptive approach involved selecting pertinent questions toward the gravitation points that seemed to emerge from our preestablished question catalog, thus augmenting the inductive explanatory insights.

All interviews were conducted by 2 authors (AYB and IS) to ensure that the same content was covered. Both interviewers were female, had received training in qualitative methods as part of their PhD studies, and had no previous relationship with any of the interviewees. All interviews took place between November 02, 2022, and May 15, 2023. Interviewees decided on the interview date and time.

Before each interview, 3 introductory slides were presented to all interviewees to ensure a common understanding of clinical dashboards, outline the underlying research questions, and clarify content-related questions. All interviews covered the following main areas: (1) general questions on dashboards and PROM usage, (2) questions about market penetration (software producers only), (3) usage of the dashboard, (4) dashboard development and data collection, (5) feature assessment, and (6) role of the patient (Multimedia Appendix 2).

We conducted the one-to-one interviews on the web (by Zoom [Zoom Video Communications] or Microsoft Teams) in English or German. During the interviews, both interviewers took notes. All interviews were audio-recorded and transcribed verbatim to perform detailed data analysis. We sent the transcripts back to the interviewees to receive approval and then anonymized all participant details. For the analysis of the interviews, we applied thematic coding to uncover potential patterns and themes within the data [31]. To identify relevant information for the design components, first, deductive coding was used to cross-validate the findings from the literature search. Second, inductive coding allowed us to identify and classify information not covered by the literature search. AYB and CW coded all interviews and conducted the analysis with Atlas.ti Windows (version 22) [32]. The study is reported in accordance with the Consolidated Criteria for Reporting Qualitative Research (COREQ) 32-item checklist to ensure transparency and reliability (Multimedia Appendix 3) [33].

### Ethical Considerations

This study was performed in line with the principles of the Declaration of Helsinki. The Ethics Committee of the University of St. Gallen was informed about this research and issued a letter of exemption.

## Results

### Overview

In total, we conducted 16 interviews with 6 software producers (I1 to I6) and 10 users (I7 to I16) until thematic saturation was reached (male=10, female=6). All software producer interviewees were from different companies and had different positions. A total of 8 out of 10 user interviewees were department heads with different specialties, such as pneumology, oncology, pediatrics, or orthopedics. This allowed us to analyze distinctive needs for design components in clinical dashboards. One of the remaining user interviewees was a chief hospital innovation officer, and the other interviewee was a member of hospital quality management. These specific interviewees lacked a formal medical background. However, based on their employment within a hospital setting, they routinely interacted closely with diverse medical practitioners and, thus, can be assumed to have a profound understanding of physicians’ needs for clinical dashboards. The software producer interviews lasted between 29 and 45 minutes, whereas the user interviews had a duration of 20 to 36 minutes (Multimedia Appendix 4).

The interviews with software producers as well as users revealed that they perceived differentiation in design depending on disease (1-time acute vs chronic condition) as not essential. Therefore, we did not distinguish between the design requirements of different disease types but instead focused on the design principles for clinical dashboards incorporating PROMs in general.

### General Information

#### Overview

To evaluate the importance of the key design components, we asked both interview groups to outline the type of disease, the setting, the type of PROM used, and the targeted key user for their clinical dashboard. Software producers mostly indicated they prefer dashboards that are applicable to chronic and acute conditions similarly (4/6) (Multimedia Appendix 5 contains the full set of codes). For users, we observed that they preferred a dashboard for either a chronic or acute disease, which often correlated with their professional focus. Both interview groups rated the outpatient setting as the preferable area of application (8/16). Both interview groups mentioned using a disease-specific PROM only (6/16) or combining generic and disease-specific PROMs (9/16). However, none of the interviewees preferred to use a generic PROM only, whereas I11 acknowledged that his team only uses generic PROMs for research purposes. Furthermore, both interview groups favored physicians (13/16), followed by other health care professionals (such as nurses or physiotherapists) (7/16) as key users. The patient was also recognized as a potential user of clinical dashboards (8/16); however, there were some discrepancies on how much and which content should be displayed to patients. I13 also highlighted that clinical dashboards may be valuable tools for relatives supporting patients in their daily activities to grasp the patient’s health status better.

#### Data Collection

The most frequently mentioned use case for data collection was for micro perspectives (ie, patient-physician communication and intrapatient comparison; 12/16), especially by users (8/10), followed by meso perspectives (ie, comparison of patient groups within departments or institutions) for both interview groups (5/16; Table 1). Furthermore, the most often mentioned purposes of reporting were “communication” (14/16) and “better basis for decision-making (for physician)” (9/16; Table 1).

For the point-in-time of data collection, there was a clear tendency (11/16) of interviewees for patients to answer the questionnaires independently at home. However, I2 mentioned that it is perceivable that the first-time data collection is conducted in the waiting room because “[patients] get an explanation by a physician or a nurse, but after that, they are on their own.” Also, interviewee I14 had a similar perception of dealing with the data collection process. For the type of data collection, a clear tendency toward web-based tools such as platforms or phone apps was indicated (10/16).

The table shows the ranking of design components’ features according to the number of mentions by software producers and users. Behind the features, the share of interviewees mentioning this design component’s feature is indicated. As the interviewees could provide more than 1 answer or no answer for each design component, shares can add up to more or less than 100%. The quantification of the responses can only be interpreted in the context of this study. However, it does not allow for generalizable statements.

**Table 1:** Overview of software producer and user responses on how data collection should be implemented in the clinical dashboard.

Software producer responses (n/N)			User responses (n/N)
**Level of reporting**			
	Micro (4/6)	Micro (8/10)	
	Macro (2/6)	Meso (4/10)	
	Meso (1/6)	Macro (1/10)	
**Purpose of reporting**			
	Improved communication (6/6)	Improved communication (8/10)	
	Better basis for physician’s decision (4/6)	Better basis for physician’s decision (5/10)	
	Real-time tracking (2/6) and shared decision-making (2/6)	Shared decision-making (2/10)	
**Data collection**			
	Digital (5/6)	Digital (5/10)	
	—^a^	Paper-based (4/10)	
**Time of data collection**			
	Independent at home (4/6)	Independent at home (7/10)	
	Before the appointment (in the waiting room) (2/6)	Before the appointment (in the waiting room) (6/10)	

^a^Not applicable.

### Dashboard Components

Concerning the dashboard components, representatives of both interview groups mentioned that some patient information (such as patient photographs, demographic information, or contact details of other care team members) needs to be presented on the dashboard (9/16). An interviewee additionally emphasized that she expects the patient information to include,

[…] key events. So, surgeries need to be shown. For example, in cancer, the start of chemotherapy, completion of chemotherapy, started radiation, that you can understand what is going on in the background of those patients.

I16

Except for I1 and I5, all other software producers rated clinical data (eg, lab results or medication data) as meaningful information that must be included in the clinical dashboard. In contrast, users tended rather not to overfill the clinical dashboard with clinical data as this information might be available from another source, as a participant mentioned:

[…] and then if they [the physicians] want to have the clinical information on the patient, they just open up the EMR [electronic medical record] and check it, which is another tab in the Chrome app.

I8

The free write-in design component did not resonate well in either of the 2 groups. A total of 6 interviewees replied that a free write-in space is not useful, whereas only 2 interviewees perceived this design component as very beneficial. Nevertheless, when having such a free write-in box included, users (3/10) wanted it as an additional source of information for some specific variables.

Software producers and users rated the past assessment PRO score as one of the most crucial design components in a clinical dashboard incorporating PROMs. From the software producer perspective, one mentioned:

This [past assessment PRO score] is very well received, simply the score progression up and down visually, so to speak.

I6

Another participant had a similar perception:

This [past assessment PRO score] is absolutely relevant because it is above all changes in these questionnaires that are significant. In any case, it is very important to look at the progression, not just the individual value.

I9

Another well-perceived design component was the peer-group comparison. All software producer interviewees (6/6) agreed on the inclusion of this feature. The users (7/10) appreciated the inclusion, too. However, 3 users did not perceive an added value in the peer-group comparison due to uncertainties in interpreting the applied PRO (I9), the missing guidance on the relevant factors to compare different patient groups (I12), or a clear reasoning on why peer group comparison is useful (I15).

Although only 1 user emphasized applying PRO-related goals, overall health-related goals were discussed more controversially within both groups. Software producer I4 reported that their dashboard includes a component where the physician can develop overall health goals together with the patient and check goal achievement, whereas I6 stated that this is not part of their dashboard. From the user perspective, this design component could add value to the patient-physician communication, as the goal statement makes the aim or expectations of the patients explicit (I9 and I16).

Concerning alerts, software producers and users have different perceptions of whether alerts should be included in the dashboard. Software producers (5/6) mentioned that an alert function needs to be included. Generally, producers were indifferent regarding whether the alert is in real time (4/6) or only appears during the appointment (4/6). A participant mentioned, however, that liability for medical risks and errors is unclear when real-time alerts are used:

[…] if [a score is] three days red or is really critical and no push notification is sent, the error is on our side. However, if a push notification is sent and the doctor does not react, the error lies with the doctor. And that is a bit of a grey area, where we still have to figure out how it's actually done.

I4

In contrast, 4 users preferred not to include alerts. I8 and I11 mentioned that this feature previously existed in their dashboard, but the acceptance was not high enough in their teams, which made them stop using it. Furthermore, I12 raised the issue that additional interpretations for physicians are required to ensure that they completely understand what the deterioration or improvement in a score means. I10 favored the alerts during the appointment to highlight critical factors and to facilitate comparison over time. I13 and I16 preferred real-time alerts allowing the treating physician to react directly to the patient’s issues.

Concerning the dashboard’s customizability, all interviewees agreed that some degree of customizability is required to meet different user needs. Although most users preferred customizability from a standard set (7/10), software producers still seemed undecided whether a clinical dashboard should be adapted to individual needs (2/6) or “off the shelf” (3/6). Nevertheless, software producers (5/6) agreed that scalability is only achievable in case clinical dashboards equipped with features defined in a standard set are provided.

### Findings From Inductive Coding

Whereas the above-presented results were derived from the deductive procedure, the subsequent inductive procedure allowed us to identify additional aspects that were not explicitly discussed in the current literature. These aspects included, on the one hand, the added value of clinical dashboards incorporating PROMs and visualization possibilities of PROMs and, on the other hand, various barriers, reducing the uptake of the clinical dashboard. Furthermore, the interviewees also discussed the patient’s access to their data controversially.

Although the visualization of the PRO scores was not extensively discussed in the literature, we asked our interviewees about their preferences. Only 2 out of 6 software producers indicated that the index and dimensional scores are always provided in their dashboards. The remaining software producers elaborated on the visualization of their PRO scores. Contrarily, almost all users (9/10) mentioned their preferences for the visualization: 4/10 users preferred to have index and dimensional scores available, 3/10 users prioritized dimensional scores, whereas 2/10 users voted for the visualization of index scores. For instance, I7 voted for the visualization of dimensional scores by stating, “*we must of course know the dimensionality and different aspects.*” In contrast, an interviewee argued:

I like [index] scores better, as I said, but because we have these individual questions like there are ten questions, and you can have a summary score of it and the system plots every question on a trend. I think that is rather messy because then you have like ten different color graphs just projected over each other, and you can click them on, or off. So it's easy, but for me it's less informative.

I11

Concerning added value provided by the clinical dashboard, software producers emphasized the visualization of results (5/6), enhanced workflow efficiency (4/6), improved data comparability (3/6), and higher patient satisfaction (3/6). Users perceived the biggest advantages of a clinical dashboard in the visualization of results (8/10), enhanced workflow efficiency (6/10), and improved data comparability (5/10). Higher patient satisfaction (2/10) was not among the most often named advantages.

Potential barriers to implementing the full potential of clinical dashboards are interoperability between various systems for both interview groups (12/16) and the burdensome collection of data for users (6/10). The reasons for the burdensome collection were various. For instance, a participant highlighted organizational issues by stating:

There is one burden that falls more on the admin staff and the secretaries, which is to make sure that the right patient is put on the right pathway with the right PROMs. So, you need to pre-program all this. Then you must either have an automatic integration saying this patient is going to see this because it needs to be personalized to the patient pathway. So how do you make sure that the right patients get the right thing without having an army of extra people?

19

Furthermore*,* a participant raised technical challenges in collecting PROs by expressing:

We had a problem for a year now that we had these computers in the waiting room and for some reason they were broken or disconnected. It took us a very long time to get them because there were all these people taking ownership of these silly computers.

I11

Furthermore, software producers mentioned legal consequences in case of displaying inadequate information (4/6) and the licensing of the PROM questionnaires (3/6) as potential barriers. Users rather perceived the lack of good data (4/10), legal consequences (3/10), and nonintuitive use (3/10) as additional barriers.

Except for 2 users (I7 and I14), all interviewees agreed that patients benefit from access to the information stored on the clinical dashboard. A total of 9 of the interviewees expect the patients to develop a better understanding and interpretation of their own health status. One software producer summarized their efforts as

[…] what we are doing now is that we have patient apps, and the patients can also see their own results in their own app. They can also say to the caregiver: ‘hey, I see this or that’, and so we involve the patients into it. I think it is important to make it patient-friendly also.

I2

Users highlighted similar scenarios, for instance:

When reading this data with PROMs, it's very clear. It's data that they have entered. If they see that they're functioning or the ability to walk a block or go on a bus is going down, they can relate to it and understand ‘Oh, actually I thought I was doing better than that’ or ‘I'm happy, actually I forgot that I couldn't and now I can.

I8

This quote also represents the statement of I14, whereas I15 additionally emphasizes the positive correlation between an enhanced understanding of one’s own health status and increased treatment adherence. Especially the score visualization supports the patient’s understanding of their own health status (6/16). Still, users (7/10) acknowledged that more explanation of the meaning of the data is required to avoid patient’s misinterpretation of the data. Furthermore, 2 users claimed that physicians and patients need different levels of aggregation of available data concerning depth of analysis and interpretation complexity to avoid overwhelming patients. Finally, I6 and I8 stated they would refuse to share clinical data with patients as clinical outcomes might frighten patients or lead to misinterpretations, such as outlined by a user:

If you look at in oncology or radio oncology to PSA levels, which are an indicator but not an actual sign of cancer, you wouldn't want the patients to learn by themselves. ‘Okay, my PSA level are up again, and I don't know what it means and what is going on.’ With this type of outcomes, you need to be very careful on how you get them across […] and this needs professional support.

I18

### Findings From Deductive and Inductive Coding

**Figure 1:**
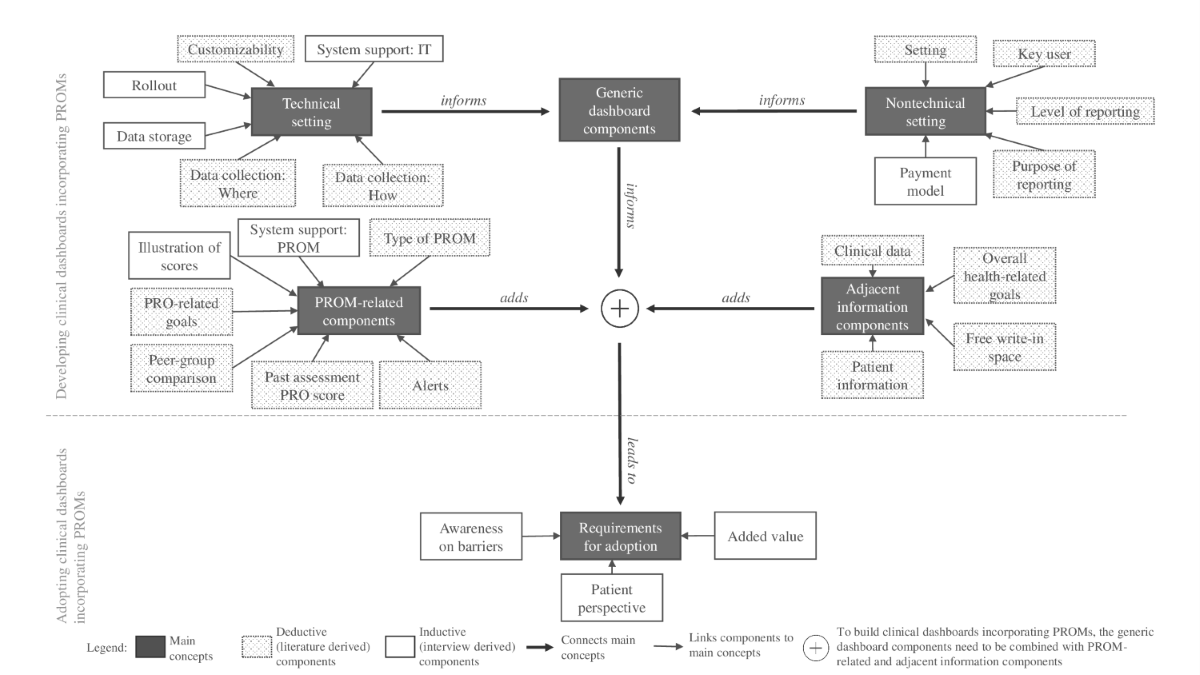
Overview on design components for developing clinical dashboards incorporating patient-reported outcome measures. PRO: patient-reported outcome; PROM: patient-reported outcome measure.

The derivation of deductive and inductive codes led to insights into how different design components are related to each other (Figure 1). To build clinical dashboards incorporating PROMs, not only the selected PROMs on the dashboard are crucial, but rather the complete construct of the clinical dashboard. In addition, generic dashboard components need to be respected as well. Furthermore, aspects such as awareness of barriers, patients’ perspectives, and the potential of added value need to be considered when reflecting on the adoption of clinical dashboards (Multimedia Appendix 6 contains a full description of all topics).

## Discussion

### Principal Findings

The qualitative research approach allowed for a more profound understanding of the design components for clinical dashboards incorporating generic and disease-specific PROMs. Our developed framework (Figure 1) displays that we found 16 design components based on a literature search and identified 9 additional design components through interviews with software producers and users. The interviews revealed that both interview groups had a similar perception of the use case of a clinical dashboard incorporating PROMs.

The use of a dashboard primarily strengthens patient-physician communication in outpatient settings. It should include a combination of disease-specific and generic PROMs. Physicians and other health care professionals were the most frequently mentioned key users. In addition, preferences for visualizing PROs, whether as index or dimensional scores, are subjective and dependent on individual inclinations. According to software producers and users, dashboards provide benefits, especially through the visualization of data and the enhancement of workflow efficiency. The main barriers to dashboard implementation were seen in the interoperability between various systems and the collection of data.

In addition, the interviews revealed that dashboard content should not vary across diseases, except by an adaptation of the disease-specific PROM. This project showed that not only design components directly displayed by the clinical dashboard incorporating PROMs but also other features, such as providing system support or the definition of key users, are relevant topics for a successful implementation.

### Comparison With Previous Work

Concerning the use of PROMs, studies show that it is common to use generic PROMs, particularly the EQ-5D, to track the health status of patients. Notably, researchers see the standardized format and content as a major benefit that facilitates its usability across different diseases and patient groups [34]. Furthermore, the multilingual questionnaire allows large-scale analyses [35,36]. Not only were disease-specific PROMs complementing generic PROMs, but their combined value was seen as more valuable than their sum, as in the case of the EQ-5D and the COPD (chronic obstructive pulmonary disease) Assessment Test (CAT) combination [37]. Interestingly, similar findings emerged from studies developing PROM dashboards for various disease areas. Baeksted et al [28] and Hassett et al [9] found it relevant to include the cancer care–specific PRO of Common Terminology Criteria for Adverse Events (CTCAE) in their dashboards, and Nicolas-Boluda et al [22] incorporated endometriosis-specific indicators into their dashboard. We found that disease-specific PROMs are preferred over generic PROMs by the interviewees. Nevertheless, a combined use of both PROMs was seen as beneficial, too.

Enhanced visualization of the patient’s PROs over time can facilitate the moderation role of the physician [12,19,38] in line with the growing demand for shared decision-making. We found a similar outcome as the interviewees perceived the purpose of a clinical dashboard incorporating PROMs in the improved communication between patient and physician. Furthermore, Desantis et al [12] found that using a clinical dashboard improves the workflow and communication of changes in the HRQoL between the patient and physician. Following this argument, interviewees emphasized that a clinical dashboard incorporating PROMs may also serve as a basis for better-informed decisions by physicians as various key information points are displayed in 1 report. Therefore, additional information besides the PRO data must be displayed to display a holistic picture of the patient’s general health conditions. Patient information (such as sociodemographic information or most recent health updates) and clinical data (such as lab results and medication lists) are required, which was previously highlighted in the literature [20,24,25] and also rated as essential by the interviewees in our study.

Furthermore, the interviewees promoted the design component “past assessment PRO score” and ranked it as one of the most important ones. Changes in the patient’s health status become apparent and traceable, and thus, enable the initiation of countermeasures immediately. Similar reasons are also provided in the literature [9,28]. Another well-perceived design component for analyzing PROs was the peer-group comparison. Interviewees claimed that this design component facilitates understanding whether a patient’s health status is above or below the norm. Hartzler et al [17] highlighted that peer-group comparison should match patients in age and treatment. In addition, Ragouzeos et al [27] advise indicating a “normal” range.

Design components where interviewees did not indicate a clear tendency were PRO-related and overall health goals and the function of alerts. Cronin et al [16] found that patients want to set and evaluate goals over time, which could be supported by a clinical dashboard. However, Liu et al [26] warned that setting goals might further pressure the patient. Furthermore, the challenge of including alerts is to decide on the appropriate alert level [39] and avoid “alert fatigue” of physicians [20].

### Limitations

Our study has 3 limitations. First, the interviewees were mainly from Western countries, especially Switzerland and Germany. A generalization of our results to other countries should, therefore, be carefully assessed. Second, the user interviewees were most often specialists, and we did not control for their years of experience. This research can be seen as a starting point in building guidance on what needs to be considered when developing clinical dashboards incorporating PROMs. Therefore, we wanted to include a broad range of opinions and needs on critical design components. Future research should consider various dashboard stakeholders from different countries with varying years of professional experience to further evaluate our proposed design components. Third, the design components were not built and evaluated in a real-world setting but only discussed during the interviews. We aimed to develop a theoretical framework of design components based on software producers’ capabilities and users’ needs. Therefore, future research approaches can build up on our findings to refine clinical dashboards and test them in clinical practice.

### Conclusions

In our study, we identified 25 design components and consolidated them into a framework of 4 main concepts. This framework strives to work as a guiding approach where researchers and practitioners can orient themselves on components to be included when building clinical dashboards incorporating PROMs. Design components such as peer-group comparison or alert thresholds require further investigation in future research, as knowledge on determining appropriate peer groups or setting alerts based on PRO thresholds needs to be improved. In addition, future research efforts may test the real-world applicability of the proposed design components by either discussing them in focus groups or implementing them in a dashboard in clinical practice.

## Appendix

Multimedia Appendix 1 Overview of design components and categories of components which laid the foundation for user and software producer interviews.

Multimedia Appendix 2 Semi-structured interview guide for software producer and user interviews.

Multimedia Appendix 3 Consolidated Criteria for Reporting Qualitative Research (COREQ).

Multimedia Appendix 4 Overview of software producer and user interviewee’s characteristics.

Multimedia Appendix 5 Output of thematic coding.

Multimedia Appendix 6 Explanation of topics from deductive and inductive coding.

## Abbreviations

CAT: COPD Assessment Test
COPD: chronic obstructive pulmonary disease
COREQ: Consolidated Criteria for Reporting Qualitative Research
CTCAE: Common Terminology Criteria for Adverse Events
HRQoL: health-related quality of life
PRO: patient-reported outcome
PROM: patient-reported outcome measure
