# Supplementary material for: A Collection of Components to Design Clinical Dashboards Incorporating Patient-Reported Outcome Measures: Qualitative Study
Source: J Med Internet Res. 2024 Oct 2;26:e55267. doi: 10.2196/55267 (PMC11483256; doi:10.2196/55267)
Supplement: Multimedia Appendix 1 [file jmir_v26i1e55267_app1.pdf]

Multimedia Appendix 1. Overview of design components and categories of components which laid the foundation for user and software producer interviews.

	Components	Categories of components	Sources
**General information**			
	Type of disease	Chronic; Acute	1–9
	Setting	Inpatient; Outpatient; Combination	1,7,10–12
	Type of PROM	Disease-specific; Generic, Combination	8,13–27
	Key user	Physician; Other health care professionals (e.g., physiotherapist, nurse, etc.); Patient; Relatives	1,9,28
**Data collection**			
	Level of reporting	Micro (patient-physician communication and intra-patient comparison); Meso (comparison of patient groups within departments or institutions); Macro (comparison of patient groups across departments or institutions)	29
	Purpose of reporting	Shared decision-making (for patient and physician); Better basis for decision (for physician); Interpretation support of data (for physician)	30–32
	Data collection	Digital; Paper-based	6,7,29
	Time of data collection	Directly during appointment; Before the appointment (in waiting room); Independent at home	6,7
**Dashboard**			
	Patient information		1,33,34
	Clinical data		1,33,35,36
	Free write-in space		10,37
	Past assessment PRO score		5,10,36,37
	Peer-group comparison		2,36,38
	PRO-related goals		3,36
	Overall health-related goals		

	Alerts	Alerts should be presented immediately on the dashboard during the appointment if there is a critical value; Alerts should be presented during the appointment; No alerts should be included	6,37
	Customizability	The dashboard components should be tailored to individual needs; The dashboard components can be chosen from a standardized set; The components of the clinical dashboard are not customizable	3,9,33

Legend: Based on a literature search, we developed a list of design components and potential categories of components to be considered when building a clinical dashboard incorporating PROMs covering three grouping areas:(1) general information, (2) data collection, and (3) dashboard components. Each grouping area includes components and categories of components. For example, in the grouping area of "general information", the component of "setting" means whether the dashboard is used in an in- or outpatient setting or in a combination of both. Additionally, for the components of "patient information", "clinical data", "free write-in space", "past assessment PRO score", "peer-group comparison", "PRO-related goals" and "overall health-related goals", the considered literature did not further specify on categories of components but only highlighted the importance of these components. These collected findings informed the development of the semi-structured interview questionnaire. Thus, these findings were thoroughly discussed during the interviews with the software producers and users.

References:

1. Rudin RS, Perez S, Rodriguez JA, et al. User-centered design of a scalable, electronic health record-integrated remote symptom monitoring intervention for patients with asthma and providers in primary care. *Journal of the American Medical Informatics Association*. 2021;28(11):2433-2444. doi:10.1093/jamia/ocab157

2. Hartzler AL, Izard JP, Dalkin BL, Mikles SP, Gore JL. Design and feasibility of integrating personalized PRO dashboards into prostate cancer care. *Journal of the American Medical Informatics Association*. 2016;23(1):38-47. doi:10.1093/jamia/ocv101

3. Cronin RM, Conway D, Condon D, Jerome RN, Byrne DW, Harris PA. Patient and healthcare provider views on a patient-reported outcomes portal. *Journal of the American Medical Informatics Association*. 2018;25(11):1470-1480. doi:10.1093/jamia/ocy111

4. Elm JJ, Daeschler M, Bataille L, et al. Feasibility and utility of a clinician dashboard from wearable and mobile application Parkinson's disease data. *NPJ Digit Med*. 2019;2(1):95. doi:10.1038/s41746-019-0169-y

5. Fautrel B, Alten R, Kirkham B, et al. Call for action: How to improve use of patient-reported outcomes to guide clinical decision making in rheumatoid arthritis. *Rheumatol Int*. 2018;38(6):935-947. doi:10.1007/s00296-018-4005-5

6. Desantis D, Baverstock RJ, Civitarese A, Crump RT, Carlson K V. A clinical perspective on electronically collecting patient-reported outcomes at the point-of-care for overactive bladder. *Canadian Urological Association Journal*. 2016;10(11-12):359. doi:10.5489/cuaj.3757

7. Nicolas-Boluda A, Oppenheimer A, Bouaziz J, Fauconnier A. Patient-reported outcome measures in endometriosis. *J Clin Med*. 2021;10(21):5106. doi:10.3390/jcm10215106

8. Heath EL, Ackerman I, Lorimer M, et al. National implementation of an electronic patient-reported outcome measures program for joint replacement surgery: Pilot study. *JMIR Form Res*. 2022;6(4):e30245. doi:10.2196/30245

9. Hartzler AL, Chaudhuri S, Fey BC, Flum DR, Lavallee D. Integrating patient-reported outcomes into spine surgical care through visual dashboards: Lessons learned from human-centered design. *eGEMs (Generating Evidence & Methods to improve patient outcomes)*. 2015;3(2):2. doi:10.13063/2327-9214.1133

10. Baeksted C, Pappot H, Nissen A, et al. Feasibility and acceptability of electronic symptom surveillance with clinician feedback using the Patient-Reported Outcomes version of Common Terminology Criteria for Adverse Events (PRO-CTCAE) in Danish prostate cancer patients. *J Patient Rep Outcomes*. 2017;1(1):1. doi:10.1186/s41687-017-0005-6

11. Tan A, Durbin M, Chung FR, et al. Design and implementation of a clinical decision support tool for primary palliative Care for Emergency Medicine (PRIM-ER). *BMC Med Inform Decis Mak*. 2020;20(1):13. doi:10.1186/s12911-020-1021-7

12. DeMellow J, Kim TY. Technology-enabled performance monitoring in intensive care: An integrative literature review. *Intensive Crit Care Nurs*. 2018;48:42-51. doi:10.1016/j.iccn.2018.07.003

13. Nolan CM, Longworth L, Lord J, et al. The EQ-5D-5L health status questionnaire in COPD: Validity, responsiveness and minimum important difference. *Thorax*. 2016;71(6):493-500. doi:10.1136/thoraxjnl-2015-207782

14. Szentes BL, Schwarzkopf L, Kirsch F, Schramm A, Leidl R. Measuring quality of life in COPD patients: Comparing disease-specific supplements to the EQ-5D-5L. *Expert Rev Pharmacoecon Outcomes Res*. 2020;20(5):523-529. doi:10.1080/14737167.2019.1662302

15. Huber MB, Kurz C, Kirsch F, Schwarzkopf L, Schramm A, Leidl R. The relationship between body mass index and health-related quality of life in COPD: real-world evidence based on claims and survey data. *Respir Res*. 2020;21(1):291. doi:10.1186/s12931-020-01556-0

16. Merino M, Villoro R, Hidalgo-Vega Á, Carmona C. Health-related quality of life of patients diagnosed with COPD in Extremadura, Spain: results from an observational study. *Health Qual Life Outcomes*. 2019;17(1):189. doi:10.1186/s12955-019-1244-4

17. Smith SMS, Jan S, Descallar J, Marks GB. An investigation of methods to improve recall for the patient-reported outcome measurement in COPD patients: a pilot randomised control trial and feasibility study protocol. *Pilot Feasibility Stud*. 2019;5(1):92. doi:10.1186/s40814-019-0475-9

18. Ayala A, Forjaz MJ, Ramallo-Fariña Y, Martín-Fernández J, García-Pérez L, Bilbao A. Response mapping methods to estimate the EQ-5D-5L from the Western Ontario McMaster Universities Osteoarthritis in patients with hip or knee osteoarthritis. *Value in Health*. 2021;24(6):874-883. doi:10.1016/j.jval.2021.01.003

19. Baghbani-Naghadehi F, Armijo-Olivo S, Prado CM, Gramlich L, Woodhouse LJ. Does obesity affect patient-reported outcomes following total knee arthroplasty? *BMC Musculoskelet Disord*. 2022;23(1):55. doi:10.1186/s12891-022-04997-4

20. Bansback N, Trenaman L, MacDonald K V., et al. An individualized patient-reported outcome measure (PROM) based patient decision aid and surgeon report for patients considering total knee arthroplasty: protocol for a pragmatic randomized controlled trial. *BMC Musculoskelet Disord*. 2019;20(1):89. doi:10.1186/s12891-019-2434-2

21. Conner-Spady BL, Marshall DA, Bohm E, Dunbar MJ, Noseworthy TW. Comparing the validity and responsiveness of the EQ-5D-5L to the Oxford hip and knee scores and SF-12 in osteoarthritis patients 1 year following total joint replacement. *Quality of Life Research*. 2018;27(5):1311-1322. doi:10.1007/s11136-018-1808-5

22. Conner-Spady BL, Marshall DA, Bohm E, et al. Reliability and validity of the EQ-5D-5L compared to the EQ-5D-3L in patients with osteoarthritis referred for hip and knee replacement. *Quality of Life Research*. 2015;24(7):1775-1784. doi:10.1007/s11136-014-0910-6

23. Eneqvist T, Nemes S, Kärrholm J, Burström K, Rolfson O. How do EQ-5D-3L and EQ-5D-5L compare in a Swedish total hip replacement population? *Acta Orthop*. 2020;91(3):272-278. doi:10.1080/17453674.2020.1746124

24. Haragus H, Prejbeanu R, Poenaru D V., Deleanu B, Timar B, Vermesan D. Cross-cultural adaptation and validation of a patient-reported hip outcome score. *Int Orthop*. 2018;42(5):1001-1006. doi:10.1007/s00264-017-3742-5

25. Rolfson O, Bohm E, Franklin P, et al. Patient-reported outcome measures in arthroplasty registries. *Acta Orthop*. 2016;87(sup1):9-23. doi:10.1080/17453674.2016.1181816

26. Sen RK, Shetti V, Mukhopadhyay R, et al. Satisfaction and health-related quality of life following hip and knee arthroplasty surgeries in Indian patients: A cross-sectional Study. *Indian J Orthop*. 2022;56(5):918-926. doi:10.1007/s43465-021-00589-x

27. Marshall DA, Jin X, Pittman LB, Smith CJ. The use of patient-reported outcome measures in hip and knee arthroplasty in Alberta. *J Patient Rep Outcomes*. 2021;5(S2):87. doi:10.1186/s41687-021-00362-6

28. Howarth M, Bhatt M, Benterud E, et al. Development and initial implementation of electronic clinical decision supports for recognition and management of hospital-acquired acute kidney injury. *BMC Med Inform Decis Mak*. 2020;20(1):287. doi:10.1186/s12911-020-01303-x

29. Watson L, Delure A, Qi S, et al. Utilizing Patient Reported Outcome Measures (PROMs) in ambulatory oncology in Alberta: Digital reporting at the micro, meso and macro level. *J Patient Rep Outcomes*. 2021;5(S2):97. doi:10.1186/s41687-021-00373-3

30. Gibbons C, Porter I, Gonçalves-Bradley DC, et al. Routine provision of feedback from patient-reported outcome measurements to healthcare providers and patients in clinical practice. *Cochrane Database of Systematic Reviews*. 2021;2021(10). doi:10.1002/14651858.CD011589.pub2

31. Berry DL, Blumenstein BA, Halpenny B, et al. Enhancing patient-provider communication with the electronic self-report assessment for cancer: A randomized trial. *Journal of Clinical Oncology*. 2011;29(8):1029-1035. doi:10.1200/JCO.2010.30.3909

32. Graupner C, Kimman ML, Mul S, et al. Patient outcomes, patient experiences and process indicators associated with the routine use of patient-reported outcome measures (PROMs) in cancer care: a systematic review. *Supportive Care in Cancer*. 2021;29(2):573-593. doi:10.1007/s00520-020-05695-4

33. Tsangaris E, Edelen M, Means J, et al. User-centered design and agile development of a novel mobile health application and clinician dashboard to support the collection and reporting of patient-reported outcomes for breast cancer care. *BMJ Surg Interv Health Technol*. 2022;4(1):119. doi:10.1136/BMJSIT-2021-000119

34. Taxter A, Johnson L, Tabussi D, et al. Co-Design of an Electronic Dashboard to Support Coproduction of Care in Pediatric Rheumatic Disease: Human-Centered Design and Usability Testing (Preprint). *J Particip Med*. Published online November 5, 2021. doi:10.2196/34735

35. Liu LH, Garrett SB, Li J, et al. Patient and clinician perspectives on a patient-facing dashboard that visualizes patient reported outcomes in rheumatoid arthritis. *Health Expectations*. 2020;23(4):846-859. doi:10.1111/hex.13057

36. Ragouzeos D, Gandrup J, Berrean B, et al. "Am I OK?" using human centered design to empower rheumatoid arthritis patients through patient reported outcomes. *Patient Educ Couns*. 2019;102(3):503-510. doi:10.1016/j.pec.2018.10.016

37. Hassett MJ, Cronin C, Tsou TC, et al. eSyM: An electronic health record–integrated patient-reported outcomes-based cancer symptom management program used by six diverse health systems. *JCO Clin Cancer Inform*. 2022;(6):1-10. doi:10.1200/cci.21.00137

38. Strachna O, Cohen MA, Allison MM, et al. Case study of the integration of electronic patient-reported outcomes as standard of care in a head and neck oncology practice: Obstacles and opportunities. *Cancer*. 2021;127(3):359-371. doi:10.1002/cncr.33272
