# Supplementary material for: A Collection of Components to Design Clinical Dashboards Incorporating Patient-Reported Outcome Measures: Qualitative Study
Source: J Med Internet Res. 2024 Oct 2;26:e55267. doi: 10.2196/55267 (PMC11483256; doi:10.2196/55267)
Supplement: Multimedia Appendix 2 [file jmir_v26i1e55267_app2.pdf]

Multimedia Appendix 2. Semi-structured interview guide for software producer and user interviews

<table>
<tr><th colspan="2">1) General questions on dashboards and PROM usage</th></tr>
<tr><td>Software Producer
1. Please describe the dashboard you are producing/designing.
2. In which countries or regions is your dashboard available?
3. What is the major goal of your clinical dashboard?
a. For what type of work (e.g., analyzing outcomes over time, communicating with the patient, comparing to other groups, getting a better overview of all data collected) is the clinical dashboard designed for?
b. What do you think is beneficial about using clinical dashboards?
c. Where do you see major barriers in using clinical dashboards?
4. Do you incorporate PROMs in your clinical dashboard? If yes, which ones (standardized sets vs. own creation)? If no, why not?
a. Does the user have the possibility to choose from a set of available PROMs or is it pre-defined by you?
b. Do you use generic and disease-specific PROMs, or just one of each? Why?
c. Why do you think it is beneficial to incorporate PROMs into clinical dashboards?
d. What do you perceive as challenging when incorporating PROMs into clinical dashboards?</td><td>User
1. What is your motivation for using clinical dashboards?
a. For what type of work (e.g., analyzing outcomes over time, communicating with the patient, comparing to other groups, getting a better overview of all data collected) do you use the clinical dashboard?
b. What do you like about the clinical dashboard you currently use?
c. What don't you like about the clinical dashboard you currently use?
2. Do you use PROMs? If yes, which ones (standardized sets vs. own creation)? If no, why not?
a. Do you use generic and disease-specific PROMs, or just one of each? Why?
b. Are PROMs already integrated into the clinical dashboard?
c. What is your motivation for using PROMs?
d. For what type of work (e.g., analyzing outcomes over time, communicating to patients, comparing to other groups) do you use PROMs?
e. What do you like about using PROMs?
f. What don't you like about using PROMs?</td></tr>
<tr><th colspan="2">2) Questions about the market (penetration)</th></tr>
<tr><td>Software Producer
1. How many clients do already use the clinical dashboards incorporating PROMs to communicate with patients?
2. How do you feel about the demand for these dashboards?
3. What designs are requested?
4. What is the general feedback from customers on your solution? What do they like - what don't they like?
5. Is there a scientific basis for the design of your dashboards? Are physicians involved in the development process?</td><td>User
Not asked to users.</td></tr>
</table>

<table>
<tr><th colspan="2">3) Usage of the dashboard</th></tr>
<tr><td>Software Producer
1. In how far does your product facilitate the workflow of practitioners?
2. Who uses and has access to the clinical dashboard?
a. Do you have a special area of expertise, or can the dashboard be used at any discipline?
b. Can it be used in an outpatient and inpatient setting?
c. Can it only be used in one department/hospital or is it conceivable that it could also be used, for example, by outpatient care providers such as primary care physicians in parallel?
3. What type of data is available in the clinical dashboard?
a. Do you receive regular feedback from your users on how valuable the included data is?
b. Do you provide support in using the clinical dashboards?
c. Do your customers own or rent the software of the clinical dashboard?
4. Do you think different episodes of care (e.g., orthopedics and COPD, one-time intervention vs chronic disease) require different dashboard capabilities? Why?</td>
<td>User
1. To what extent does using the clinical dashboard make your day-to-day work easier?
2. Who uses and has access to the clinical dashboard?
a. Is it only for use in your department/hospital or is it conceivable that it could also be used, for example, by outpatient care providers such as primary care physicians?
3. Is all the data you need in your clinical practice included in the clinical dashboard?
a. What type of data is available in the clinical dashboard?
b. In your opinion, is there a principle missing that you would find particularly valuable?
c. What addition did you notice when using the clinical dashboard for the first time?
d. Did you feel that using a clinical dashboard was complicated and could be made easier in the future?
e. Do you find that using the clinical dashboard for the first time was intuitive?
i. Have you received any support, or have you familiarized yourself with its use?
4. Do you think different episodes of care (e.g., orthopedics and COPD) require different dashboard capabilities? Why?</td></tr>
<tr><th colspan="2">4) Dashboard development and data collection</th></tr>
<tr><td>Software Producer
1. How do you develop a dashboard for a client? Do you have a basic product that is customizable to different needs, or do you develop it every time from scratch according to the needs of your client?
a. How would you describe your working mode? Do you work agile or in a waterfall structure?
b. How many iteration cycles do you go through until the delivery of the final product?
2. How does the roll-out of a new clinical dashboard work?
3. How does the data collection work? (Where,</td>
<td>User
1. Who provides the dashboard you use?
a. How was it developed?
b. Is it customizable to individual needs?
2. How was the clinical dashboard implemented in your organization?
3. How does the data collection work? (Where, when, how, who, ...)
a. Where are difficulties in the data collection?</td></tr>
</table>

<table>
<tr><td>when, how, who, ...)
a. Where do you see difficulties in data collection?
4. Where is the data stored?
a. Do you have access to the data collected by your customers?
5. Is the collected data also used for other purposes than for the improvement in the patient-physician communication?
a. E.g., aggregation of data and comparison between hospitals, other research purposes, etc.</td><td></td></tr>
<tr><td colspan="2">5) Dashboard Component Assessment</td></tr>
<tr><td colspan="2">Same questions for software producers and users
From the literature, we have extracted some design principles that could be included in clinical dashboards - please provide your opinion on displaying these design principles and whether you already incorporate these design principles in your clinical dashboard:
1. Past PROM assessments (including evolution over time).
a. What kind of scores should the dashboard display? Values for individual dimensions or index scores? Why?
2. Future PROM-related goals
3. Overall health-related goals
4. Benchmarking | peer-group comparison
5. Alerts on symptom/symptom change warnings
a. If yes, at what time should the alarm/warning appear?
6. Patient information (patient photo, demographic information, recent health updates, and contact information for other care team members)
7. Clinical data (laboratory results and drug data)
a. What medical data do you use?
8. Free write-in area
9. In your opinion, is a design principle missing?</td></tr>
<tr><td colspan="2">6) Role of the patient</td></tr>
<tr><td>Software Producer
1. What is the role of the patient?
a. Does the patient also have access to the information on the dashboard?
b. Do you assist the patient in interpreting the data?
c. Do you think the patient's needs concerning the dashboard differ whether he/she underwent a one-time intervention or whether he/she suffers from a chronic disease?
d. To what extent is the information different from that displayed to the health professional/physician?</td><td>User
1. What is the role of the patient?
a. Does the patient also have access to the information on the dashboard?
b. Should the patient be assisted in interpreting the information?
c. To what extent is the information different from that displayed to the health professional/physician?</td></tr>
</table>

End
Is there anything else you would like to add to conclude the interview?
