# Supplementary material for: A Collection of Components to Design Clinical Dashboards Incorporating Patient-Reported Outcome Measures: Qualitative Study
Source: J Med Internet Res. 2024 Oct 2;26:e55267. doi: 10.2196/55267 (PMC11483256; doi:10.2196/55267)
Supplement: Multimedia Appendix 3 [file jmir_v26i1e55267_app3.pdf]

Multimedia Appendix 3. Consolidated Criteria for Reporting Qualitative Research (COREQ)

No. Item	Guide questions/description	Reported on Page #
**Domain 1: Research team and reflexivity**		
Personal Characteristics		
1. Interviewer/facilitator	Which author/s conducted the interview or focus group?	Page 4
2. Credentials	What were the researcher's credentials? E.g. PhD, MD	Page 4
3. Occupation	What was their occupation at the time of the study?	Page 4
4. Gender	Was the researcher male or female?	Page 4
5. Experience and training	What experience or training did the researcher have?	Page 4
Relationship with participants		
6. Relationship established	Was a relationship established prior to study commencement?	Page 4
7. Participant knowledge of the interviewer	What did the participants know about the researcher? e.g. personal goals, reasons for doing the research	Page 4
8. Interviewer characteristics	What characteristics were reported about the interviewer/facilitator? e.g. Bias, assumptions, reasons and interests in the research topic	Page 4
**Domain 2: study design**		
Theoretical framework		
9. Methodological orientation and Theory	What methodological orientation was stated to underpin the study? e.g. grounded theory, discourse analysis, ethnography, phenomenology, content analysis	Page 4
Participant selection		
10. Sampling	How were participants selected? e.g. purposive, convenience, consecutive, snowball	Page 4
11. Method of approach	How were participants approached? e.g. face-to-face, telephone, mail, email	Page 4
12. Sample size	How many participants were in the study?	Page 5
13. Non-participation	How many people refused to participate or dropped out? Reasons?	n/a
Setting		

14. Setting of data collection	Where was the data collected? e.g. home, clinic, workplace	Page 4
15. Presence of non-partici-pants	Was anyone else present besides the partici-pants and researchers?	No
16. Description of sample	What are the important characteristics of the sample? e.g. demographic data, date	Pages 4&5, Multimedia Appendix 4
Data collection		
17. Interview guide	Were questions, prompts, guides provided by the authors? Was it pilot tested?	Page 4
18. Repeat interviews	Were repeat interviews carried out? If yes, how many?	No
19. Audio/visual recording	Did the research use audio or visual recording to collect the data?	Page 4
20. Field notes	Were field notes made during and/or after the interview or focus group?	Page 4
21. Duration	What was the duration of the inter views or focus group?	Page 5, Multimedia Ap-pendix 4
22. Data saturation	Was data saturation discussed?	Page 4
23. Transcripts returned	Were transcripts returned to participants for comment and/or correction?	Page 4
**Domain 3: analysis and find-ings**		
Data analysis		
24. Number of data coders	How many data coders coded the data?	Page 5
25. Description of the coding tree	Did authors provide a description of the cod-ing tree?	Page 10 (Figure 1), Mul-timedia Appendix 5
26. Derivation of themes	Were themes identified in advance or derived from the data?	Page 5
27. Software	What software, if applicable, was used to manage the data?	Page 5
28. Participant checking	Did participants provide feedback on the find-ings?	No
Reporting		
29. Quotations presented	Were participant quotations presented to illus-trate the themes/findings? Was each quotation identified? e.g. participant number	Pages 7-10
30. Data and findings con-sistent	Was there consistency between the data pre-sented and the findings?	Yes

31. Clarity of major themes	Were major themes clearly presented in the findings?	Pages 7-10, Multimedia Appendix 5
32. Clarity of minor themes	Is there a description of diverse cases or discussion of minor themes?	Pages 7-10, Multimedia Appendix 5
