# Supplementary material for: A Collection of Components to Design Clinical Dashboards Incorporating Patient-Reported Outcome Measures: Qualitative Study
Source: J Med Internet Res. 2024 Oct 2;26:e55267. doi: 10.2196/55267 (PMC11483256; doi:10.2196/55267)
Supplement: Multimedia Appendix 4 [file jmir_v26i1e55267_app4.pdf]

Multimedia Appendix 4. Overview of software producer and user interviewee's characteristics

Interview ID	Software producer/ User (gender)	Function	Country	Interview duration
I1	Software producer (male)	Product Owner	Netherlands	45 mins
I2	Software producer (female)	Product Delivery Manager	Netherlands	43 mins
I3	Software producer (male)	Digital strategy lead	Switzerland	29 mins
I4	Software producer (male)	CEO & founder	Germany	36 mins
I5	Software producer (male)	Director Smart Health Academy & Patient Reported Outcomes	Germany	44 mins
I6	Software producer (female)	Chief Medical Officer	Germany	33 mins
I7	User (male)	Chief physician at department of orthopedics	Switzerland	29 mins
I8	User (male)	Chief hospital innovation officer	Switzerland	28 mins
I9	User (male)	Chief physician at department of pneumology	Switzerland	30 mins
I10	User (female)	Member of hospital quality management	Switzerland	34 mins
I11	User (male)	Chief physician at department of pediatrics and rheumatology	Netherlands	36 mins
I12	User (male)	Chief physician at department of pneumology	Switzerland	35 mins
I13	User (female)	Chief physician at department of orthopedic surgery and trau-matology	Switzerland	34 mins
I14	User (female)	Chief physician at department of orthopedics	Germany	21 mins
I15	User (male)	Chief physician at department of pneumology	Germany	20 mins
I16	User (female)	Chief physician at department of cancer care	USA	24 mins
