# Supplementary material for: A Collection of Components to Design Clinical Dashboards Incorporating Patient-Reported Outcome Measures: Qualitative Study
Source: J Med Internet Res. 2024 Oct 2;26:e55267. doi: 10.2196/55267 (PMC11483256; doi:10.2196/55267)
Supplement: Multimedia Appendix 5 [file jmir_v26i1e55267_app5.pdf]

Multimedia Appendix 5. Output of thematic coding

	Software Producer (SP)						User (U)										Interview guide	
	**I1**	**I2**	**I3**	**I4**	**I5**	**I6**	**I7**	**I8**	**I9**	**I10**	**I11**	**I12**	**I13**	**I14**	**I15**	**I16**	**SP**	**U**
**Added value**	**X**	**X**	**X**	**X**	**X**	**X**	**X**	**X**	**X**	**X**	**X**		**X**	**X**	**X**	**X**	**1.3a-b & 1.4c**	**1.1a-b & 1.2c-e**
- comparability			X	X		X		X			X		X	X		X		
- efficiency	X	X		X		X	X		X	X	X				X	X		
- improvements						X	X							X		X		
- patient satisfaction		X			X	X		X								X		
- visualisation		X	X	X	X	X	X	X		X	X		X	X	X	X		
**Alert**		**X**	**X**	**X**	**X**	**X**		**X**	**X**	**X**	**X**	**X**	**X**	**X**	**X**	**X**	**5.5**	**5.5**
- during appointment		X	X		X	X				X				X	X			
- no								X	X		X	X						
- real time		X	X	X	X								X			X		
**Awareness on barri-ers**	**X**	**X**	**X**	**X**	**X**	**X**	**X**	**X**	**X**	**X**	**X**	**X**	**X**	**X**	**X**	**X**	**1.3c & 1.4d**	**1.1c & 1.2f**
- burdensome collec-tion of PROs		X					X	X			X	X		X		X		
- interoperability		X	X	X		X	X	X	X	X	X	X		X		X		
- lack of good data							X			X			X	X				
- lack of motivation of users	X	X														X		
- legal consequences	X	X	X	X			X			X	X							
- licensing of ques-tionnaires		X			X	X												
- not intuitive to use								X							X	X		
- various interests							X		X									
**Clinical data**		**X**	**X**	**X**		**X**		**X**	**X**	**X**	**X**	**X**	**X**	**X**	**X**	**X**	**5.7**	**5.7**
- clinical data very useful		X	X	X		X			X				X	X	X			
- not included								X		X	X	X				X		
**Customizability**		**X**		**X**	**X**	**X**	**X**	**X**		**X**	**X**	**X**	**X**	**X**	**X**	**X**	**4.1**	**4.1b**
- from a standard set		X			X	X	X	X		X	X		X	X		X		

	Software Producer (SP)						User (U)										Interview guide	
	**I1**	**I2**	**I3**	**I4**	**I5**	**I6**	**I7**	**I8**	**I9**	**I10**	**I11**	**I12**	**I13**	**I14**	**I15**	**I16**	**SP**	**U**
- to individual needs				X	X							X			X			
**Iterative dashboard development**	**X**	**X**	**X**			**X**		**X**		**X**	**X**		**X**			**X**	**4.1b**	**4.1a**
**Data storage**			**X**	**X**	**X**						**X**						**4.4**	**4.3**
- At producer			X		X													
- Cloud			X	X														
- in clinic				X							X							
**Different needs btw diseases**			**X**	**X**	**X**	**X**		**X**		**X**				**X**			**3.4**	**3.4**
- no				X	X	X		X		X				X				
- yes			X															
- different needs in various countries				X														
**Data collection: How**	**X**	**X**		**X**	**X**	**X**	**X**		**X**	**X**	**X**	**X**	**X**	**X**		**X**	**4.3**	**4.3**
- digital	X	X		X	X	X	X			X	X		X			X		
- paper-based									X			X	X	X				
**Data collection: Where**	**X**	**X**		**X**	**X**	**X**	**X**		**X**	**X**	**X**	**X**	**X**	**X**		**X**	**4.3**	**4.3**
- in waiting room		X				X	X			X	X	X	X	X		X		
- independent at home	X	X		X	X				X	X	X	X	X			X		
**Free write-in area**		**X**		**X**	**X**	**X**		**X**	**X**	**X**	**X**	**X**	**X**	**X**	**X**	**X**	**5.8**	**5.8**
- not useful					X	X		X		X			X			X		
- neutral														X				
- rather useful																		
- very useful		X													X			
- yes, but specific question				X		X			X		X	X						
**Key user**	**X**	**X**	**X**	**X**	**X**	**X**	**X**	**X**	**X**		**X**	**X**	**X**	**X**		**X**	**3.2**	**3.2**
- physician	X	X	X	X	X	X	X		X		X	X	X	X		X		

	Software Producer (SP)						User (U)										Interview guide	
	I1	I2	I3	I4	I5	I6	I7	I8	I9	I10	I11	I12	I13	I14	I15	I16	SP	U
- other health care professionals	X	X			X	X	X		X							X		
- patient			X	X	X	X		X				X	X			X		
- relatives													X					
**Level of reporting**	**X**	**X**		**X**	**X**	**X**	**X**	**X**	**X**	**X**	**X**	**X**		**X**		**X**	**1.3 & 4.5a**	**1.1a**
- macro	X	X												X				
- meso	X						X	X			X			X				
- micro		X		X	X	X	X	X	X	X	X	X		X		X		
**Overall health related goals**	**X**	**X**	**X**	**X**	**X**	**X**		**X**	**X**		**X**	**X**	**X**	**X**		**X**	**5.3**	**5.3**
- less useful														X				
- neutral									X							X		
- not included		X			X	X		X				X	X					
- rather useful																		
- very useful	X		X	X							X							
**Past assessment score**	**X**	**X**		**X**		**X**	**X**	**X**	**X**	**X**	**X**	**X**	**X**	**X**	**X**	**X**	**5.1**	**5.1**
- very useful	X	X		X		X	X	X	X	X	X	X	X	X		X		
- not useful															X			
**Patient information**		**X**	**X**	**X**	**X**	**X**		**X**		**X**	**X**	**X**	**X**	**X**	**X**	**X**	**5.6**	**5.6**
- no					X			X		X			X					
- yes		X	X	X		X					X	X		X	X	X		
**Patient perspective**		**X**	**X**	**X**	**X**	**X**	**X**	**X**	**X**	**X**	**X**	**X**	**X**	**X**	**X**	**X**	**6.1**	**6.2**
- all PRO results shown		X		X				X										
- better interpretation of own health status		X	X		X			X	X	X			X	X		X		
- clinical data not to be shown						X		X										

	Software Producer (SP)						User (U)										Interview guide	
	**I1**	**I2**	**I3**	**I4**	**I5**	**I6**	**I7**	**I8**	**I9**	**I10**	**I11**	**I12**	**I13**	**I14**	**I15**	**I16**	**SP**	**U**
- different processing of data for physician and patient															X	X		
- more explanation on the meaning of the data		X		X	X			X		X		X				X		
- no							X							X				
- no additional help for PROM fill-out		X						X		X								
- visualisation of scores		X			X				X	X	X					X		
**Payment model**			**X**	**X**	**X**	**X**	**X**										**3.3c**	**(not explicitly covered in the interview guide)**
- add-on to product (for free)			X				X											
- license				X	X	X	X											
**Peer-group comparison**	**X**	**X**	**X**	**X**	**X**	**X**	**X**	**X**	**X**	**X**	**X**	**X**	**X**	**X**	**X**	**X**	**5.4**	**5.4**
- no									X			X			X			
- yes	X	X	X	X	X	X	X	X					X	X		X		
- yes, but currently not possible										X	X							
**PRO-related goals**		**X**		**X**	**X**	**X**		**X**			**X**	**X**	**X**	**X**	**X**	**X**	**5.2**	**5.2**
- neutral																X		
- not included		X		X	X			X			X	X	X		X			
- rather useful						X												
- very useful														X				
**Purpose of reporting**	**X**	**X**	**X**	**X**	**X**	**X**	**X**	**X**	**X**		**X**	**X**	**X**	**X**		**X**	**1.3**	**1.1**

	Software Producer (SP)						User (U)										Interview guide	
	**I1**	**I2**	**I3**	**I4**	**I5**	**I6**	**I7**	**I8**	**I9**	**I10**	**I11**	**I12**	**I13**	**I14**	**I15**	**I16**	**SP**	**U**
- better basis for physician's decision		X	X		X	X		X	X		X	X	X					
- improved communication	X	X	X	X	X	X	X	X	X		X	X	X	X		X		
- real-time tracking			X	X														
- shared decision-making				X		X	X	X										
**Roll-out**				**X**		**X**	**X**		**X**	**X**				**X**			**4.2**	**4.2**
- step by step (in different clinics)				X		X	X		X	X								
- top down										X				X				
**Scores**				**X**		**X**	**X**	**X**	**X**	**X**	**X**	**X**	**X**	**X**		**X**	**5.1**	**5.1**
- both				X		X		X			X			X		X		
- dimensional							X			X			X					
- index									X			X						
**Setting**		**X**	**X**	**X**	**X**		**X**		**X**	**X**	**X**	**X**	**X**	**X**	**X**	**X**	**3.2b**	**3.2**
- combination					X					X					X	X		
- inpatient							X											
- outpatient		X	X	X					X		X	X	X	X				
**System support: IT**		**X**	**X**	**X**	**X**				**X**	**X**			**X**			**X**	**3.3b**	**3.3d & 3.3e**
- in-house at corresponding institution									X									
- introduction		X	X						X	X						X		
- no					X								X					
- workshops/webinars			X													X		
**System support: PROM**		**X**	**X**	**X**						**X**				**X**			**3.3b**	**3.3d-e**

	Software Producer (SP)						User (U)										Interview guide	
	**I1**	**I2**	**I3**	**I4**	**I5**	**I6**	**I7**	**I8**	**I9**	**I10**	**I11**	**I12**	**I13**	**I14**	**I15**	**I16**	**SP**	**U**
- interpretation of PRO scores		X	X	X						X				X				
**Type of disease**	**X**	**X**	**X**	**X**	**X**		**X**		**X**		**X**	**X**	**X**	**X**	**X**	**X**	**1.1**	**1.1 (Derived from specialization)**
- chronic		X							X		X				X	X		
- both	X		X	X	X							X	X					
- one-time intervention							X							X				
**Type of PRO**	**X**	**X**	**X**	**X**	**X**	**X**	**X**	**X**	**X**	**X**	**X**	**X**	**X**	**X**		**X**	**1.4b**	**1.2a**
- combination		X		X	X	X	X			X		X	X	X				
- disease-specific	X		X					X	X		X					X		
- generic - only for research											X							

Legend: SP = Software producer, U = User, btw = between

The last column referring to the interview guide (for more information, please see Supplementary Appendix 2) indicates which question corresponds to the thematic coding output. Exemplarily, for “type of PRO” questions 1.4b in the software producer guide and 1.2a in the user guide are applicable. This means that the question is allocated to “1) General questions on dashboards and PROM usage” and refers to the specific question 2 respectively 4 in the corresponding software producer and user guide. Section 5 of the interview guide assessed the different dashboard components. Therefore, in this coding output, we display the assessment categorically of how valuable the interviewees rate the individual components. This categorization applies to the following components: past assessment score, PROM-related goals, overall health-related goals, peer-group comparison, patient information, clinical data, and free write-in area.
