# Supplementary material for: A Collection of Components to Design Clinical Dashboards Incorporating Patient-Reported Outcome Measures: Qualitative Study
Source: J Med Internet Res. 2024 Oct 2;26:e55267. doi: 10.2196/55267 (PMC11483256; doi:10.2196/55267)
Supplement: Multimedia Appendix 6 [file jmir_v26i1e55267_app6.pdf]

Multimedia Appendix 6. Explanation of topics from deductive and inductive coding

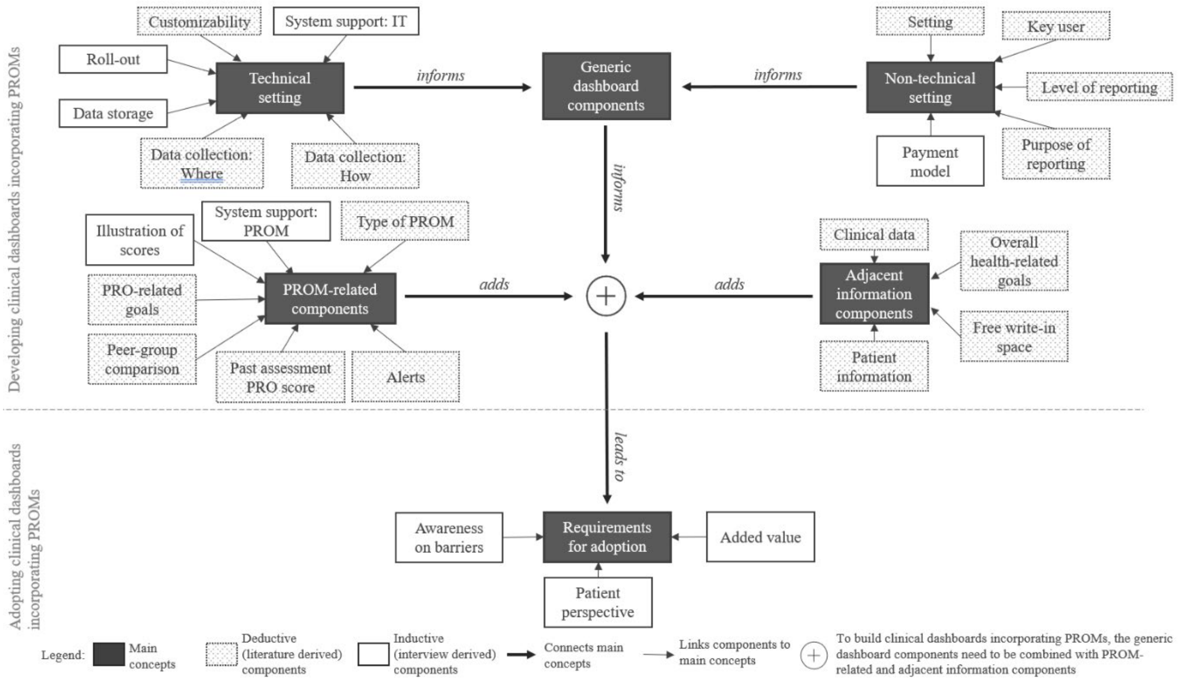

The list of 25 design principles indicates what needs to be considered when developing a clinical dashboard incorporating PROMs. This appendix provides a guiding question and a non-conclusive list of potential features for each design principle.

Technical setting:

- **System support: IT*:** How does the software producer support the system use?
    - E.g., in-house support by the corresponding institution, introduction to the system use, workshop and webinars, or no support
- **Customizability:** What degree should customizability be possible/allowed?
    - E.g., customizability to individual needs, customizability from a standard set, or no customizability
- **Roll-out*:** How should the roll-out of the clinical dashboard be conducted?
    - E.g., step-by-step or top-down
- **Data storage*:** Where should the data be stored?
    - E.g., at software producer, in a cloud solution, in the corresponding institution (i.e., clinic)
- **Data collection: Where:** When/Where should the collection take place?
    - E.g., directly during the appointment, before the appointment in the waiting room, or independent at home
- **Data collection: How:** How should the data collection take place?
    - E.g., digital or paper-based

Non-technical setting:

- **Setting:** In which setting is the clinical dashboard used?
  - E.g., inpatient, outpatient, or combination
- **Key user:** Who is the key user of the clinical dashboard?
  - E.g., all kind of physicians, other health care professionals (such as nurses or physiotherapists), patients, relatives, or third parties (such as hospital quality manager)
- **Level of reporting:** What is the preferred/chosen level of reporting?
  - E.g., micro (patient-physician communication and intra-patient comparison), meso (comparison of patient groups within departments or institutions), or macro (comparison of patient groups across departments or institutions)
- **Purpose of reporting:** For which activity is the data collected? What does the clinical dashboard serve for?
  - E.g., shared decision-making, better basis for decision, improved communication, or real-time tracking
- **Payment model*:** How does the payment model of the clinical dashboard look like?
  - E.g., add-on to another product (i.e., for free) or license

PROM-related components:

- **Type of PROM:** What kind of PROMs are used in the clinical dashboard?
  - E.g., disease-specific (such as CAT, WOMAC, HOOS, KOOS, etc.), generic (such as EQ-5D, PROMIS, WHO-5, etc.), or combination
- **System support: PROM*:** Does the software producer provide any support in analysing or interpreting the PROM data?
- **Illustration of scores*:** How are the PRO scores visualized?
  - E.g., index, dimensions, or both
- **PRO-related goals:** Are PRO-related goals included in the dashboard?
- **Peer-group comparison:** Is a peer-group comparison possible?
- **Past assessment PRO score:** Can the evolution of the PRO scores be tracked over time?
- **Alerts:** Should alerts be incorporated in/displayed by the clinical dashboard?
  - E.g., immediately when critical values appears, during appointment, or no alerts

Adjacent information components:

- **Clinical data:** Is clinical data such as lab results or medication information included in the dashboard?
- **Overall health-related goals:** Are overall health related goals included in the dashboard?
- **Free write-in space:** Is it possible to note additional information?
    - E.g., yes, only to specific questions/items, or not possible
- **Patient information:** Is patient information such patient photograph, demographic information, or recent health updates included in the dashboard?

Requirements for adoption:

- **Awareness on barriers*:** What potential barriers might hinder the adoption of clinical dashboards?
    - E.g., burdensome collection of PROs, interoperability, lack of good data, lack of motivation of users, legal consequences, licensing of questionnaires, dashboard not intuitive to use, various interest of different stakeholders
- **Patient perspective*:** What data should be provided to the patient?
    - E.g., all PRO results shown, better interpretation of own health status, clinical data not to be shown, different processing of data for physician and patient, more explanation on the meaning of the data, no additional help for PROM fill-out, visualization of scores, no data to be provided
- **Added value*:** What value can the use of a clinical dashboard incorporating PROMs add?
    - E.g., comparability between PRO scores, efficiency in workflow, improvements for physicians performance (personal learning), enhanced patient satisfaction, visualization of data

*all marked topics are derived by inductive coding, i.e., are interview-based.
